# How Emerging and Re-Emerging Infectious Diseases Challenge Our Understanding of Viral Infections

**DOI:** 10.3390/vaccines12121339

**Published:** 2024-11-28

**Authors:** My K. Ha

**Affiliations:** 1 Center for Health Economics Research and Modelling Infectious Diseases (CHERMID), Vaccine and Infectious Disease Institute, University of Antwerp, 2610 Wilrijk, Belgium; my.ha@uantwerpen.be; 2 Antwerp Center for Translational Immunology and Virology (ACTIV), Vaccine and Infectious Disease Institute, University of Antwerp, 2610 Wilrijk, Belgium; 3 Antwerp Unit for Data Analysis and Computation in Immunology and Sequencing (AUDACIS), University of Antwerp, 2020 Antwerp, Belgium

The twenty-first century has witnessed a wave of severe infectious disease outbreaks, having a devastating impact on lives and healthcare systems around the globe. The 2003 severe acute respiratory syndrome coronavirus (SARS-CoV-1) outbreak, the 2009 swine flu pandemic, the 2012 Middle East respiratory syndrome coronavirus outbreak, the 2013–2016 Ebola virus epidemic in West Africa, the 2015 Zika virus outbreak, and the recent 2019 severe acute respiratory syndrome coronavirus 2 (SARS-CoV-2) pandemic all resulted in substantial morbidity and mortality while spreading viruses across borders to infect people in multiple countries [1,2,3]. Although medical advances, improved sanitation, and the development of novel vaccines/therapeutics have reduced overall fatality, some infectious diseases such as HIV/AIDS, tuberculosis, and malaria remain a substantial burden in developing countries due to inequalities in access to affordable healthcare [4]. Moreover, deaths from emerging and re-emerging infections, in comparison with seasonal and endemic infections, have persisted throughout the twenty-first century [5]. This highlights a potential new era of infectious diseases, defined by outbreaks of emerging, evolved, re-emerging, and endemic pathogens that spread and behave unlike those the world has previously encountered.

This Special Issue, titled “Epidemiology, Virology and Prevention of Infectious Diseases in Humans”, in *Vaccines*, together with the equivalent Joint Special Issue in *Life*, aims to explore the nature of human viruses, the epidemiology of human viral diseases, their pathogenesis, diagnosis, and treatment, and disorders of host immune responses through basic and translational research. For the two joint Special Issues, we have received nine contributions, including four research articles, two reviews, two brief reports, and one communication article. Five papers report findings on SARS-CoV-2 infection and the COVID-19 pandemic, four of which are population-based epidemiological studies. Saleh et al. characterized the evolution of SARS-CoV-2 infection amongst youth in Los Angeles during 2020–2022. They noted that most youths had asymptomatic or mild COVID-19 and that underlying pulmonary conditions increased the risk of severe COVID-19. A review on the reality of influenza and COVID-19 management in Nigeria was contributed by Kabantiyok et al., who believed that the avian influenza outbreak provided Nigeria with a certain level of preparedness that was useful during the COVID-19 pandemic, although the effect of such experience is hindered by the paucity of empirical research data. AlBahrani et al. identified a high body mass index as an independent risk factor associated with an increased risk of lower oxygen saturation, lung infiltrate, and mortality, which in turn increased the need for mechanical ventilation and intensive medical care. Therefore, they suggested that individuals with obesity should be closely monitored when hospitalized for COVID-19. Martens et al. studied the IgG antibody responses to two types of COVID-19 vaccines: the Pfizer BNT162b2, the Moderna mRNA-1273, and the combination of them. They analyzed more than 20,000 samples acquired in Manitoba and identified how factors such as age and the type of vaccine can influence antibody responses to vaccination, as well as how antibody titers wane over time. Rahman et al. found that SARS-CoV-2 infection paralyzed perforin expression in the early stage and suggested using serum perforin as a potential marker for identifying the acute phase of COVID-19. Relevant to SARS-CoV-2 infection, Sechan et al. discovered that nasal IgA could be part of a fast acute response to endemic HCoV infection, caused by the human coronaviruses NL63, 229E, OC43, and HKU1, and may play a role in clearing the infection. Apart from studies about SARS-CoV-2 and other human coronaviruses, we also received two contributions discussing HIV infection. da Silva Góes et al. demonstrated a high rate of exposure to *C. trachomatis* in newly diagnosed HIV-infected individuals in the Amazon region of Brazil and suggested that all PLHA should be screened for *C. trachomatis* to decrease transmission of the bacteria and prevent the clinical manifestations of chronic infection. Hokello et al. contributed a review providing new insights into the HIV life cycle, the possible mechanism for the Th1 to Th2 shift during HIV infection, and the preferential infection of Th2 cells during the late symptomatic stage of HIV disease. We also received a contribution from Fu et al., who found that JAT could ameliorate murine norovirus (MNV)-induced NLRP3-N-GSDMD-dependent pyroptosis by inhibiting the MAPKs/NF-κB signaling pathways and decrease MNV replication in RAW264.7 macrophages, which suggests that JAT could be a potential candidate as a therapeutic agent for treating norovirus gastroenteritis.

A changing world necessitates using a novel scientific approach to assess future risks associated with infectious diseases. This opens up new research opportunities focusing on concurrent changes, examining how shifts in demographics, climate, and technology may collectively influence the emergence of pathogens and the dynamics of their global spread. There is a need for more proactive studies that explore potential future scenarios, alongside the retrospective analyses that currently dominate the field. As progress is made against longstanding endemic infections, the institutional frameworks designed to tackle these historical challenges can be repurposed to address emerging threats. Furthermore, future research must adopt a global perspective on disease risk. In our increasingly interconnected world, the risk of infectious diseases is shared globally. The COVID-19 pandemic, with its rapid worldwide spread of evolved strains, underscores the need for a collaborative international framework for research on and the control of infectious diseases. Responding to disease outbreaks will remain a significant challenge, particularly in resource-limited countries. Therefore, there is a pressing need for both localized and global control strategies that consider current socioeconomic and psychosocial conditions, along with an effective healthcare system.
